# False positive angiographic aneurysm of the anterior segment of the M1 bifurcation of the middle cerebral artery: a case report

**DOI:** 10.3389/fneur.2023.1327878

**Published:** 2023-12-20

**Authors:** Wen Xiao, Xiaolin Hou, Dingjun Li, Dongdong Yang

**Affiliations:** ^1^The Department of Neurology, Hospital of Chengdu University of Traditional Chinese Medicine, Chengdu, China; ^2^The Department of Neurosurgery, Sichuan Provincial People’s Hospital, University of Electronic Science and Technology of China, Chengdu, China; ^3^The Department of Neurosurgery, Hospital of Chengdu University of Traditional Chinese Medicine, Chengdu, China

**Keywords:** middle cerebral artery (MCA), occluded stump, false-positive aneurysm, CTA, MRA, DSA

## Abstract

Occlusion of an intracranial arterial branch, resulting in a false positive aneurysm on vascular imaging, is extremely rare, with only a few reports in the literature and mostly in the posterior circulation artery or the middle cerebral artery (MCA) bifurcation. We report a case of a 69 years-old woman with a subacute infarct lesion in the left frontal lobe, for whom both computed tomographic angiography (CTA) and digital subtraction angiography (DSA) of the cerebral vessels showed aneurysms in the anterior segment of the M1 bifurcation of the middle cerebral artery (MCA) and in the bifurcation of the MCA. The aneurysm in the MCA bifurcation was found during craniotomy, whereas the anterior segment of the M1 bifurcation had intact branch vessels with severe atherosclerosis and no aneurysm was present. The branch vessel of M1 was presumed to be atherosclerotic occlusion resulting in the distal vessels without contrast filling on CTA and DSA, and only the occluded stump at the beginning of the vessel was filled with contrast, showing an aneurysm-like morphology, which was very confusing. This case highlights to neurologists that the diagnosis of aneurysm by cerebrovascular CTA or DSA must be carefully differentiated to avoid misdiagnosis, especially if the unruptured aneurysm is in an uncommon location in combination with ischemic cerebrovascular disease.

## Introduction

Occlusion of an intracranial arterial branch, resulting in a false positive aneurysm on vascular imaging, is extremely rare, with only a few reports in the literature and mostly in the posterior circulation artery or the MCA bifurcation (1–3). A so-called false positive aneurysm is actually a stump of a proximal artery following a distal arterial occlusion. To expand the clinical diagnostic thinking of neurologists, we report a rare case of a false positive angiographic aneurysm of the anterior segment of the M1 bifurcation of the MCA combined with a true aneurysm in the MCA bifurcation.

False positive aneurysms at the M1 bifurcation of the middle cerebral artery (MCA) are rare. Neurologists should be cautious when diagnosing aneurysms using computed tomography angiography (CTA) or digital subtraction angiography (DSA). It is important to differentiate accurately to avoid misdiagnosis, especially for unruptured aneurysms in rare locations associated with ischemic cerebrovascular disease. Magnetic resonance imaging (MRI) fusion techniques may be helpful in the differential diagnosis of false positive aneurysms.

## Case report

**Figure 1:**
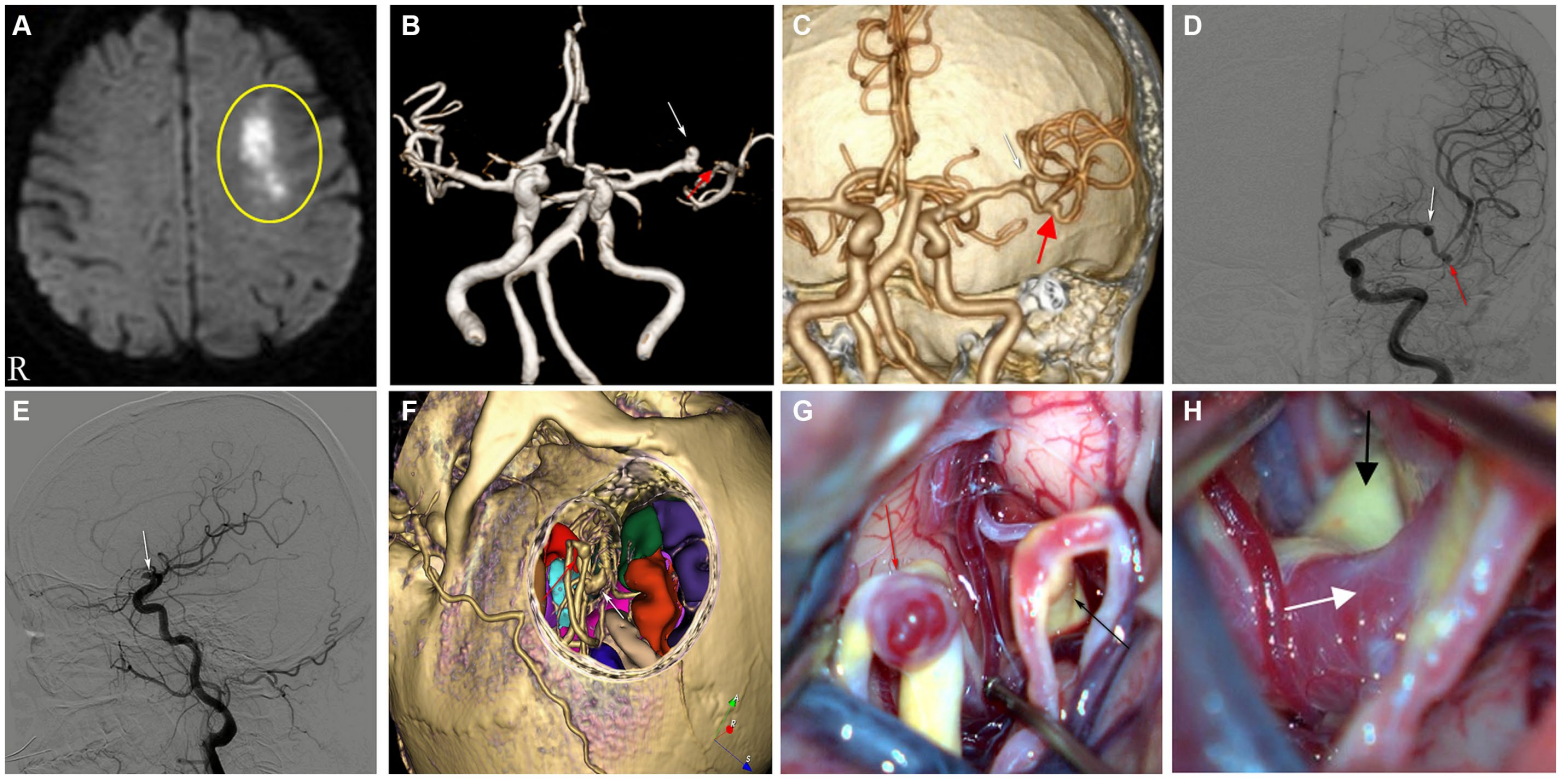
**(A)** DWI showing acute infarct lesion in the anterior segment of the left frontal hemi-oval center. **(B)** MRA showing aneurysm of the left MCA-M1 bifurcation anterior segment (white arrow) and MCA bifurcation showing a defect (red arrow). **(C,D)** CTA and DSA (orthostatic), aneurysm of the anterior segment of the M1 bifurcation of the left MCA (white arrow), MCA bifurcation aneurysm (red arrow). **(E)** DSA (lateral), aneurysm of the anterior segment of the M1 bifurcation of the left MCA (white arrow). **(F)** Preoperative surgical simulation showing aneurysm of the left MCA-M1 bifurcation anterior segment (white arrow), showing aneurysm of the left MCA bifurcation (red arrow). **(G)** Presence of an intact vessel branch in the M1 bifurcation anterior segment with significant atherosclerosis (black arrow) and aneurysm of the left MCA bifurcation (red arrow). **(H)** The left MCA-M1 bifurcation anterior segment trunk with marked atherosclerosis (black arrow), the branch vessel initiation segment there, which is a false positive aneurysm, shown by cerebrovascular imaging (white arrow).

A 69 years-old woman with a history of hyperlipidemia suddenly developed dysphasia and left-sided facial palsy during a walk 1 week previously and was hospitalized because her symptoms continued to worsen. Diffusion-weighted imaging (DWI) revealed a subacute infarct lesion in the left frontal lobe (Figure 1A). CTA of cerebral vessels revealed a cystic aneurysm in the anterior segment of the M1 bifurcation of the left MCA, approximately 3.6 mm × 4.2 mm and a cystic irregular aneurysm in the MCA bifurcation, approximately 4.7 × 4.8 mm (Figure 1C). Magnetic resonance angiography (MRA), DSA (Figures 1D,E), and CTA findings were consistent, but the MCA bifurcation showed a defect in the MRA (Figure 1B). Considering that the patient had two aneurysms in the MCA, especially the risk of rupture of the cystic irregular aneurysm in the MCA bifurcation, a craniotomy was performed to clip the aneurysms. The 3Dslicer and Freesurfer software (4) were used to simulate the surgical approach before the operation to clearly show the location and morphology of the abovementioned two aneurysms (Figure 1F).

During surgery, after separating the lateral fissure vein, the MCA bifurcation aneurysm was exposed and clipped (Figure 1G). We continued to investigate along the posterior part of the M1 bifurcation and found a branch of the M1 bifurcation with an intact vessel in the anterior part of the M1 bifurcation, which showed obvious atherosclerotic changes, and the beginning of the vessel here was misdiagnosed as an aneurysm before surgery, which was very confusing (Figures 1G,H). After repeated confirmation and careful judgment, we diagnosed that the aneurysm shown on angiography should be a vascular stump after stenosis or occlusion caused by atherosclerosis in the distal vessel, and not an aneurysm, so no further operation was performed.

After surgery, she was discharged from the hospital after 2 weeks of medication and presented with residual mild symptoms of speech disfluency. At the 6 months follow-up, the patient exhibited significant recovery, demonstrating restored speech function, and was maintained on long-term oral anticoagulants and lipid-lowering agents.

## Discussion

A meta-analysis that included 68 studies of 94,912 patients from 21 countries found that the overall prevalence of unruptured intracranial aneurysms was approximately 3.2% in people without other comorbidities (5). Another systematic evaluation including 12,781 patients with a definite diagnosis of TIA/light stroke yielded a prevalence of 5.1% for unruptured intracranial aneurysms (6). From this, we hypothesized that the probability of intracranial aneurysm is higher in patients with combined ischemic cerebrovascular disease than in patients without ischemic cerebrovascular disease; therefore, in this case, cerebrovascular imaging was performed, and two aneurysms were found.

This case had many unusual features, including the clinical presentation, the structural features of the arterial occlusion, and the anatomy of the MCA branches and combined MCA-M1 bifurcation and MCA bifurcation aneurysms, which contributed to the initial misdiagnosis. Spontaneous intracranial subarachnoid hemorrhage induced by a ruptured middle cerebral artery aneurysm is common, and the choice of craniotomy or endovascular intervention is safe and effective, but the latter has a higher rate of asymptomatic thromboembolic events, more frequently in the setting of acute subarachnoid hemorrhage (7). To date, no reports of false positive aneurysms on angiography have been reviewed in the literature on spontaneous subarachnoid hemorrhage.

In contrast, a meta-analysis of unruptured MCA aneurysms shows that a surgical clipping aneurysm remains highly safe and effective. The efficacy and safety of endovascular treatment of unruptured MCA aneurysms continue to improve; however, it has a low occlusion rate (8).

MRA, CTA, and DSA are commonly used to detect intracranial aneurysms. One study found that the sensitivity of MRA for detecting intracranial aneurysms was 95% and the specificity was 89%, false negative and false positive aneurysms detected on MRA were mainly located at the skull base and MCA (9). At present, DSA is commonly used internationally as the “gold standard” for the diagnosis of intracranial aneurysms, especially 3D-DSA, which has high sensitivity and specificity for the diagnosis of small aneurysms (10). Therefore, cerebrovascular CTA and DSA are more accurate in the detection of intracranial aneurysms. In this case, cerebrovascular CTA and DSA were performed successively, both of which revealed the presence of two aneurysms: left MCA-M1 bifurcation and MCA bifurcation aneurysms. However, during the craniotomy, we found that the patient’s left MCA-M1 bifurcation, which was actually a branch vessel of the MCA, showed severe atherosclerotic changes, and during the disease progression, the vessel was occluded, so both CTA and DSA failed to provide us with the whole vessel morphology, and the angiography only showed the morphology of the stump after vessel occlusion, which looked very much like an aneurysm, thus leading to misdiagnosis.

Vascular stumps that are misdiagnosed as aneurysms are mostly located in the posterior circulation and MCA. Only one case report of a cerebral angiography of a 70 years-old man revealed an aneurysm at the intersection of the anterior communicating artery and the A1-A2 segment of the right anterior cerebral artery, which was found to be a tapered duplication of the A1 segment of the right anterior cerebral artery by craniotomy and, thus a false positive aneurysm (11).

To the best of our knowledge, there are no reports of false positive aneurysms on angiographic imaging of the MCA-M1 bifurcation, and our case is the first of its kind. Yu et al. (3) reported on a 57 years-old man with recurrent right-sided weakness and aphasia, who was diagnosed with an aneurysm at the left MCA bifurcation on both CTA and DSA, with Moyamoya phenomenon in the vicinity. However, the “aneurysm” was found to be the stump of an occluded vessel during craniotomy. Lee et al. (1) reported a 26 years-old man with recurrent left-sided hemiparesis and an aneurysm at the right MCA bifurcation on both CTA and DSA. However, three bifurcations of the middle cerebral artery with normal upper and lower trunk vessels and significant atherosclerosis in the middle trunk branch vessels were found during craniotomy, so it was postulated that the “aneurysm” shown in the imaging was only the stump of the middle trunk branch after occlusion. Park et al. (2) reported two cases of occluded vessel stumps at MCA bifurcations that showed false positive aneurysms on angiography. Case 1 was a 52 years-old woman with no symptoms; a left middle cerebral artery aneurysm was found by MRA and CTA, along with moyamoya phenomenon near the aneurysm. Case 2 was a 62 years-old woman with years of non-specific headaches and mild hypertension; a right middle cerebral artery aneurysm was found by MRA and DSA. The false positive aneurysms reported above were all diagnosed intraoperatively as vascular stumps.

In addition, there were two cases of MCA aneurysms diagnosed by angiography that were confirmed to be vascular stumps after conservative treatment (12, 13). In one of these cases, after the patient had to opt for conservative treatment for personal reasons, it was detected on a review of the cerebral angiogram that the pre-treatment diagnosis was incorrect (12). These are the rare cases that can be searched so far, indicating the lack of awareness among neurologists that intracranial vascular stumps may present as aneurysmal patterns on vascular imaging.

In summary, there is a total of six cases of false positive middle cerebral artery aneurysms reported prior to our report, with patients undergoing DSA angiography with vascular stumps located at the bifurcation or trifurcation of the middle cerebral artery. Four of these cases had symptoms of cerebral ischemia, and the other two cases were found during screening for cerebrovascular disease. In contrast, the false positive aneurysm (vascular stump) in our case was located in the anterior segment of the M1 bifurcation of the MCA, a highly confusing location, and interestingly, there was also a true aneurysm at the M1 bifurcation of the MCA, which could easily be misdiagnosed as a multiple aneurysm of the MCA.

This case highlights that the traditional cerebral vascular imaging tools, namely, MRA, CTA, and DSA, are not absolutely reliable for the diagnosis of intracranial aneurysms. Neurologists need to take into account that atherosclerosis or arterial entrapment can lead to occlusion or stenosis of the vessel and the formation of a vascular stump, resulting in a false positive aneurysm on angiography. If such patients are misdiagnosed and endovascular interventional embolization or craniotomy is performed, it may lead to adverse outcomes.

Kuribara et al. (14) utilized 3D-fast imaging employing steady-state acquisition (FIESTA) and MRA fusion imaging on the occluded MCA’s distal segment, revealing vascular details beyond the occlusion, up to the M3 segment. Similarly, Ozaki et al. (15) combined 3D-T2 sampling perfection with application-optimized contrast using different flip angle evolution (SPACE) and MRA, providing a visualization of the occluded artery by merging the flow void effect in T2-SPACE with its MRA image. These MR fusion techniques may be instrumental in delineating obstructed vessels before mechanical thrombectomy (MT) for acute cerebrovascular occlusions. In addition, high-resolution vessel wall imaging (HRVWI) has been used to evaluate intracranial vascular pathologies such as intracranial atherosclerosis, occlusion, Moyamoya disease, vasculitis, and reversible cerebral vasoconstriction syndrome, and is useful for evaluating the vessel wall in the presence of stenosis (16).

In our case, if the abovementioned examinations had been perfected preoperatively, the branch stenosis originating from the anterior segment of the M1 bifurcation of the MCA may have been detected, thus avoiding misdiagnosis of it as an aneurysm. A literature search revealed that there are no relevant studies utilizing the abovementioned examinations as a means of identifying false positive aneurysms in vascular stumps.

## Conclusion

While infrequent, false positive aneurysms identified by vascular imaging warrant consideration by neurologists. This is particularly pertinent in cases where patients exhibit unruptured aneurysms in atypical locations concomitant with other ischemic cerebrovascular pathologies, including cerebral infarction or Moyamoya disease. A judicious selection of therapeutic strategies is imperative. In addition, MR fusion techniques may be helpful in the differential diagnosis of false positive aneurysms.

## Data availability statement

The original contributions presented in the study are included in the article/supplementary material, further inquiries can be directed to the corresponding author.

## Ethics statement

The studies involving humans were approved by Hospital of Chengdu University of Traditional Chinese Medicine. The studies were conducted in accordance with the local legislation and institutional requirements. The participants provided their written informed consent to participate in this study. Written informed consent was obtained from the individual(s) for the publication of any potentially identifiable images or data included in this article.

## Author contributions

WX: Writing – original draft. XH: Writing – original draft. DL: Conceptualization, Data curation, Writing – review & editing. DY: Supervision, Writing – review & editing.
